# Novel homozygous mutation in the FANCA gene (c.2222G>A) in a Chinese girl of Fanconi anemia

**DOI:** 10.1002/pdi3.63

**Published:** 2024-03-17

**Authors:** Wen Xianhao, Qin Hongcheng, Liao Meiling, Guan Xianmin

**Affiliations:** ^1^ Department of Hematology and Oncology Children’s Hospital of Chongqing Medical University Chongqing China; ^2^ National Clinical Research Center for Child Health and Disorders Chongqing China; ^3^ Ministry of Education Key Laboratory of Child Development and Disorders Chongqing China; ^4^ Chongqing Key Laboratory of Pediatrics Chongqing China; ^5^ Department of Pediatrics The Second Affiliated Hospital of Army Medical University Chongqing China

**Keywords:** *FANCA *gene, Fanconi anemia, next‐generation sequencing

## Abstract

Fanconi anemia is the most common inherited bone marrow failure syndrome. Its clinical manifestations include congenital dysplasia, bone marrow hematopoietic failure and tumor susceptibility. At present, there are 23 related gene abnormalities, among which *FANCA* is the most common. We report a case of a Chinese girl with bone dysplasia and aplastic anemia. The next‐generation sequencing results showed a homozygous mutation in the *FANCA* gene (c.2222G > A), which was predicted to be a pathogenic mutation based on protein function. This mutation at this site has not been reported in the previous literature. The diagnosis of Fanconi anemia should be determined with combined clinical, chromosome breakage test and gene sequencing results.

## 1 BACKGROUND

Fanconi anemia (FA) is the most common inherited bone marrow failure syndrome (IBMFS) caused by gene abnormalities and is characterized by multiple congenital malformations, bone marrow failure, and a high predisposition to hematological and solid cancers. The incidence of FA is calculated to be approximately 1 to 5 cases in 1 million births. To date, 23 Fanconi and Fanconi‐like DNA repair genes have been identified, which have an autosomal recessive inheritance pattern, except *FANCB* (X‐linked recessive) and *FANCR* (autosomal dominant).^1^ The most common gene is *FANCA*, which accounts for approximately 60∼65% of cases, followed by *FANCC* (10–15%), *FANCG* (∼10%), and *FANCD2* (3–6%); these genes harbor homozygous or double heterozygous mutations. Most FA patients carry individual mutations, resulting in a large heterogeneous list of *FANCA* mutations, including large deletions; small indels; and nonsense, splicing, frameshift and missense mutations, as reported in the databases. Here, we report a novel homozygous mutation (c.2222G > A) in exon 24 of the *FANCA* gene identified in a Chinese girl.

## 2 CASE PRESENTATION

**FIGURE 1:**
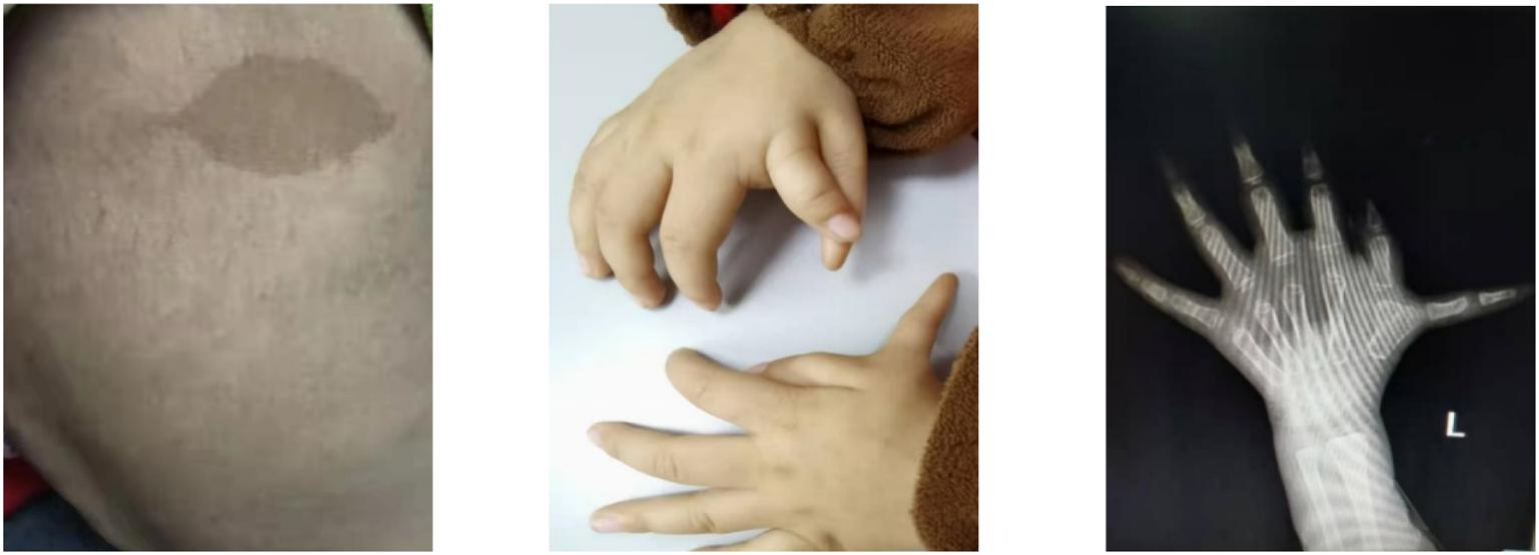
Abnormal pigmentation (café‐au‐lait) and Polydactyly.

A girl from Yunnan Province in Southwest China presented with short stature and pancytopenia at 2 years and 1 month old. She developed pancytopenia when she was 10 months old. Over time, she had progressive aggravation of pancytopenia (Table 1). She needed transfusion of red blood cells and platelets and sometimes presented with fever and cough. She had pale skin with abnormal pigmentation (café‐au‐lait), micrognathia, and polydactyly (Figure 1). There was no hepatosplenomegaly or lymphadenopathy. She was born prematurely at 35^+6^ gestational weeks and had a birthweight of 2100 g. At admission, her height was 82 cm and her weight was 9 kg. The parents were not in a consanguineous marriage, and both were in good health without hematological diseases or tumors.

**TABLE 1:** Blood routine tests at different age.

Age	White blood cell	Neutrophil	Hemoglobin	Platelet	Reticulocyte
	(*10^9^/L)	(*10^9^/L)	(g/L)	(*10^9^/L)	(%)
10 m	3.2	0.8	82	25	0.7%
1 y 2 m	3.5	0.72	78	24	‐
2 y 1 m	3.2	0.57	53	9	0.3%
2 y 4 m^a^	3	0.6	68	22	0.32%

^a^ After 3 months treatment with stanozolol.

**FIGURE 2:**
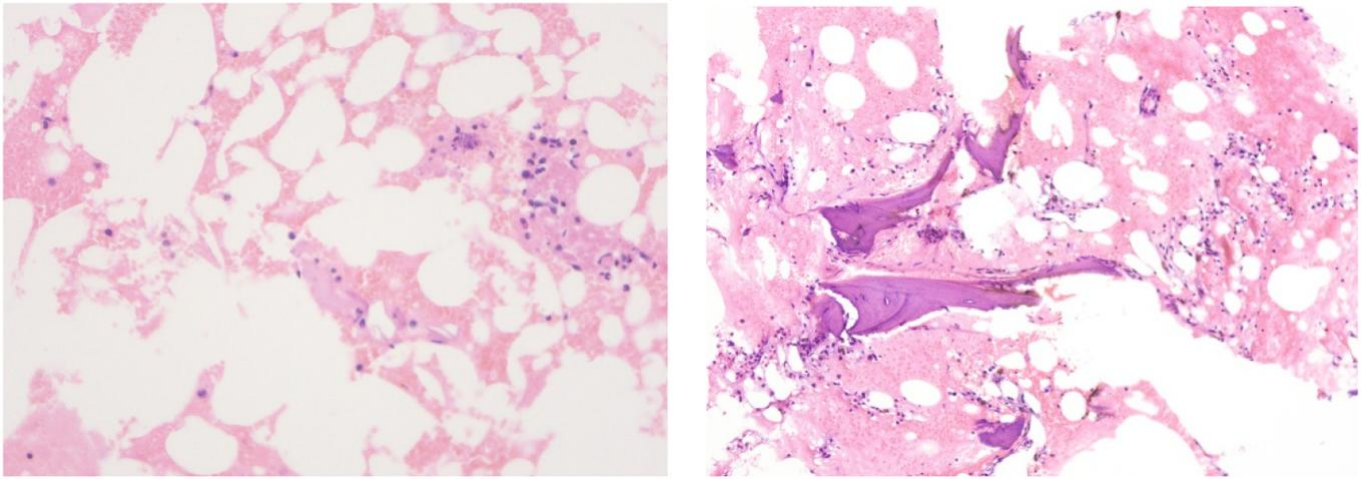
Bone marrow biopsy revealed hypocellular marrow with loss of megakaryocytes and megakaryoblasts.

**FIGURE 3:**
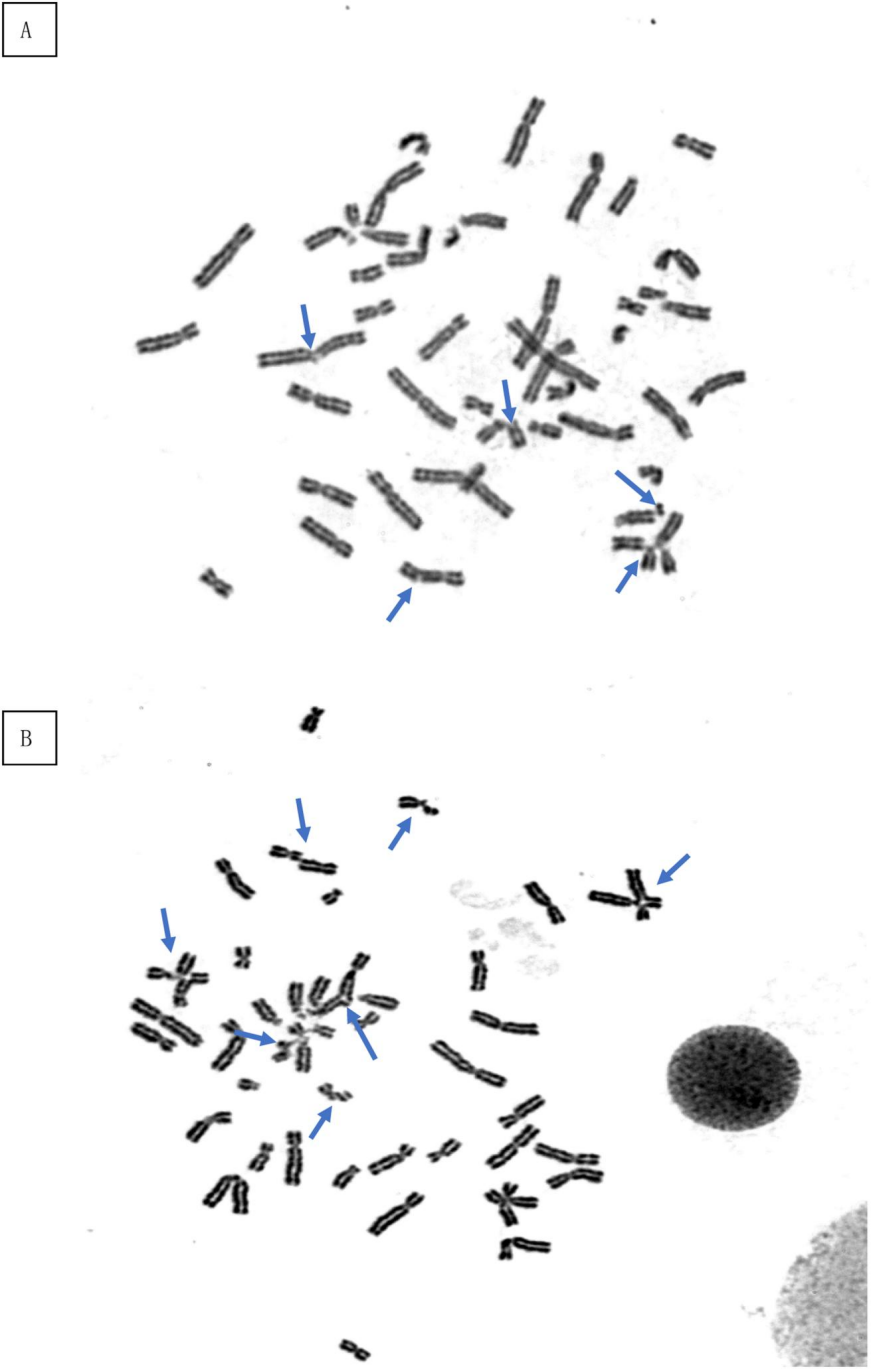
Chromosome breakage test by MMC induction (A), 50ng/mL; (B), 100ng/mL.

**FIGURE 4:**
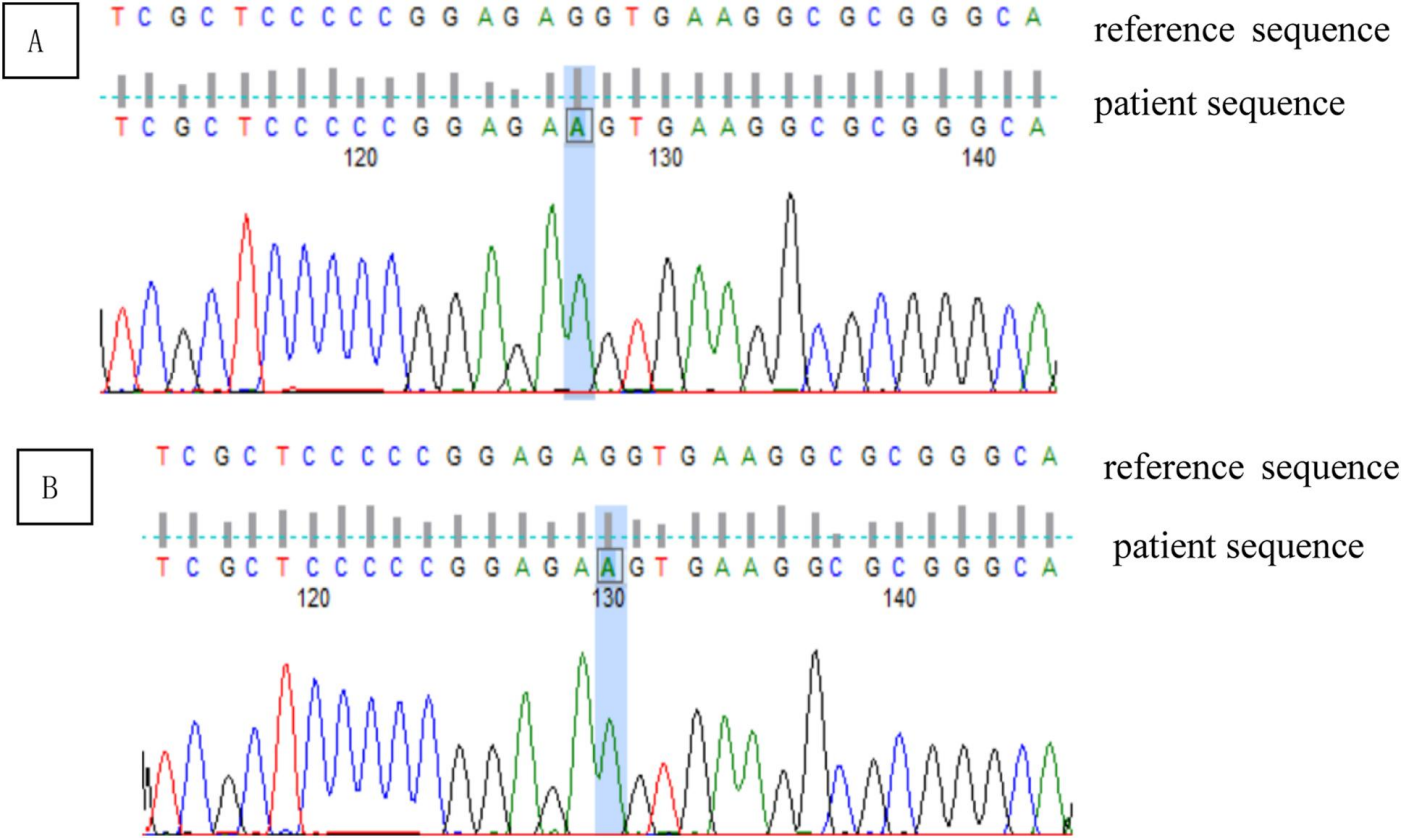
First generation verification of patient (A), peripheral blood; (B), throat swab.

**FIGURE 5:**
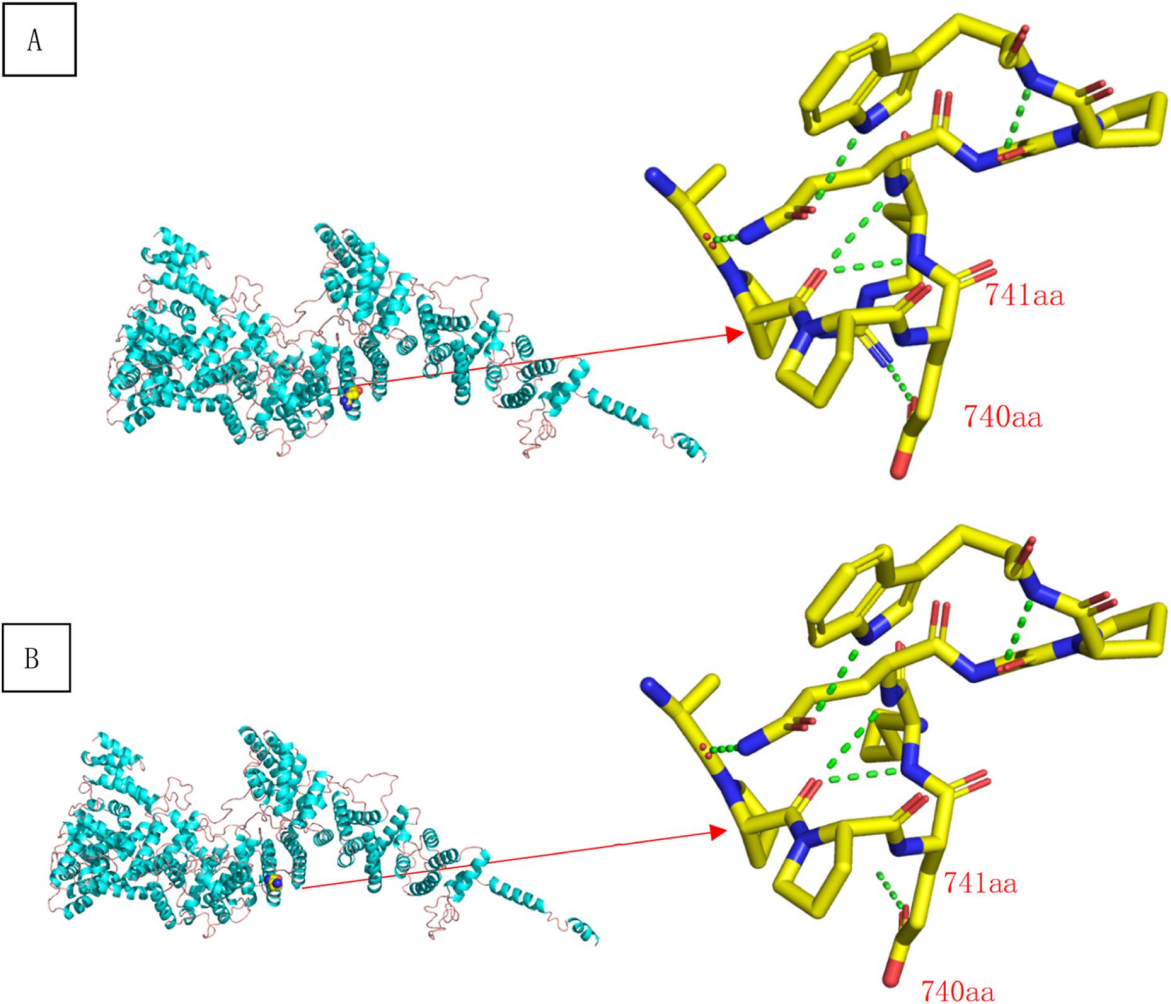
The structural analysis of Mut FANCA protein. (A), wild type; (B), mutant.

At the early stages at the local hospital, the bone marrow smear showed hyperplastic marrow with megakaryocyte mature hindrance. At admission, the bone marrow smear and biopsy revealed hypocellular marrow with loss of megakaryocytes and megakaryoblasts (Figure 2). There was severe DNA damage in peripheral blood lymphocytes and the percentage of comet cells was 25%. The chromosome breakage test by mitomycin C induction was positive, with a fracture rate of 38.0% at 50 ng/mL (control, 0.0%–9.0%) and 272.0% at 100 ng/ml (control, 0.0%–32.0%) (Figure 3). The NGS (next generation sequencing) analysis results were confirmed by direct DNA sequencing of the *FANCA* gene for the homozygous mutation c.2222G > A (p.Arg741Lys) in exon 24 (Figure 4). Direct sequencing showed that both parents of the patient were carriers of the *FANCA* c.2222G > A (p.Arg741Lys) heterozygous mutation. This variation is not included in the gnomAD (Genome Aggregation Database) and there are no relevant literature reports. According to ACMG guidelines, this variation is of unknown clinical significance. The SWISS‐MODEL database was used to determine the 3D model of the wild‐type FANCA protein and predict the protein changes caused by the amino acid changes. The structural analysis of Mut FANCA protein shows that the identified mutation (p.Arg741Lys) breaks the hydrogen bond between amino acid 740 and amino acid 741, leading to a decrease in the number of hydrogen bonds, which may affect the protein structure (Figure 5).

Therefore, based on its progressive development of pancytopenia, bone marrow failure, congenital developmental abnormalities, positive chromosome breakage test, and homozygous gene mutations, the patient was diagnosed with Fanconi anemia caused by a novel homozygous mutation in the *FANCA* gene, c.2222G > A (p.Arg741Lys), which is a pathogenic mutation. It was suggested to undergo hematopoietic stem cell transplantation, but the parents refused. After receiving oral treatment with stanozolol for 3 months, there was no change in hematopoiesis. She also needed transfusion and then missed follow‐up.

## 3 DISCUSSION

Fanconi anemia is the most common hereditary bone marrow failure disease, which causes aplastic anemia and usually occurs in childhood. Because of its various clinical manifestations, the diagnosis of Fanconi anemia is usually delayed. Many patients are diagnosed only when they progress to bone marrow failure or tumor.^2^ In this case, the reason why she went to the hospital was due to decreased blood cells rather than congenital developmental abnormalities. However, if there was no combined congenital developmental abnormalities, she was also prone to missed diagnosis. Therefore, for patients with congenital developmental abnormalities and pancytopenia, long‐term follow‐up and monitoring should be conducted. Due to genomic instability, FA cells are very sensitive to cross‐linking agents, such as mitomycin C (MMC) and dicycloxybutane (DEB). The chromosome breakage test has been widely used in the diagnosis of FA. However, due to possible somatic mosaicism and other genetic diseases, chromosome breakage tests may produce false‐negative or false‐positive results. For FA patients without obvious clinical manifestations, molecular diagnosis is necessary if the chromosome breakage test is negative. In populations with a high incidence rate of FA and in blood relatives, gene sequencing technology is an important method for diagnosing FA. With the continuous development of molecular diagnostic technology, the genetic version of Fanconi anemia was first confirmed in the 1990s. To date, 23 Fanconi and Fanconi‐like DNA repair genes have been identified. The most common gene is *FANCA*, which is located on chromosome 16q24.3, contains 43 exons along a coding sequence of 4.3 kb and spans approximately 80 kb.^3^, ^4^ Large deletions are a frequent subtype, especially in *FANCA*, accounting for 20%–40% of all pathogenic variants of this gene.^5^

This patient had a homozygous mutation in the *FANCA* gene (c.2222G > A) which was predicted as a pathogenic mutation by protein function, and this mutation has not been reported in the past. Although it was a previously unreported site, the final diagnosis of the patient was Fanconi anemia with protein function prediction software, clinical manifestations and positive chromosome breakage test.

When patients with Fanconi anemia experience hematopoietic failure, the only radical cure is hematopoietic stem cell transplantation. If there is no chance for hematopoietic stem cell transplantation, androgen treatment can be considered, meanwhile side effects such as liver damage need to be followed up. *FANCA* is an autosomal recessive inheritance, and both parents of the girl have heterozygous mutations. If it is necessary to have another child, prenatal genetic testing is recommended.

## AUTHOR CONTRIBUTIONS

Xianhao Wen managed the patients' care and wrote the manuscript; Hongcheng Qin and Meiling Liao performed the data collection and managed the patients' care; Xianmin Guan helped perform the analysis with constructive discussions. Xianhao Wen revised the manuscript. All authors read and approved the final manuscript.

## CONFLICT OF INTEREST STATEMENT

The authors declare that they have no conflicts of interest.

## ETHICS STATEMENT

The study protocol was approved on 21 February 2023 by the institutional ethics committee (Institutional Review Board of Children's Hospital of Chongqing Medical University).

## CONSENT FOR PUBLICATION

The parents of the patient provided written informed consent to publish the case report and accompanying images.

## Data Availability

Research data are not shared.
